# Accessing medical care in the era of the digital revolution: arguing the case for the “*digitally marginalised*”

**DOI:** 10.3389/fdgth.2024.1468633

**Published:** 2024-10-21

**Authors:** Anoop C. Choolayil, Sadhishkumar Paranthaman, Vijesh Sreedhar Kuttiatt

**Affiliations:** Unit of Clinical and Molecular Medicine, ICMR-Vector Control Research Centre, Puducherry, India

**Keywords:** digital divide, digitalised health, older adults, health equity, digital literacy, digital marginalisation

## Abstract

This article explores the intersection of healthcare accessibility and digitalisation from a rights perspective. Drawing from two illustrative cases presented to a filariasis management clinic in Puducherry, where the authors are affiliated, the article argues that despite the multiple benefits that digital health poses, there are individuals and sections of society that experience marginalisation in healthcare owing to digitalisation. Collating the data generated through the observations of the authors and the narratives of the patients, the article illustrates that such marginalisation can originate even from a relatively simple ICT adaptation like text message-based appointments, inducing health inequities. The impact of such digital marginalisation disproportionately affects vulnerable sections like older adults and the rural population in an intersectional pattern where disadvantages compound to produce larger health inequities for the affected. The study advocates for bridging the digital divide through efforts including digital literacy–when possible–and alternative solutions like dedicated helpdesks, training healthcare staff and involving NGOs and voluntary organisations to ensure health equity for the digitally marginalised.

## Introduction

The digitalisation of services has improved the efficacy in many sectors, including healthcare, benefitting the common people. Digital health initiatives enable patients to access information and make informed choices regarding their health (1). In fact, technologies are rapidly digitalising healthcare in what is currently known as the healthcare industry, which postulates health as a service industry and the patients as consumers of this service, the benefits being reduced operating costs and efficacious health units (2). However, the digital revolution resulted in an intangible and abstract social division, viz., the digital divide (3). The first level digital divide exists between the individuals who have access to the internet and those who do not, and the second level is between those able to use the internet competently and those who cannot (4). As Information and Communication Technology (ICT) gets increasingly integrated into the various facets of social life, there emerges a third level in terms of inequalities in the advantages gained by ICT (5). Over the years, this led to a new social division, viz. that of digital citizens and digital immigrants (3). State mechanisms have been rolling out digital literacy programmes to close down the digital divide. For instance, despite having three major digital literacy programmes dating back to 2014 (6, 7), India still lags behind with only 38 per cent of the households digitally literate (8). The increasing digitalisation of public services, despite underwhelming outputs from digital literacy efforts, exerts a disproportionate impact on the “digitally marginalised”. For instance, evidence from research on the ICT usage of older adults suggests that they are increasingly being sidelined from society due to the overwhelming digitalisation of services (4, 9). Unfortunately, pre-existing disparities influence the adoption and utilisation of these technologies, often leaving behind disadvantaged groups (10). In a certain way, digitalisation itself leads to the “marginalisation” of a section of society, as digital illiteracy is a disabling condition that restricts individuals from attaining their full potential.

The digitalisation of healthcare was aimed at facilitating comprehensive and personalised healthcare (11). While ICT innovations improved the healthcare system in many aspects, access and skills in using digital technologies became a new major determinant of health and health care. In the context of India, one of the most basic applications of digitalisation of health services is associated with mHealth–a subset of eHealth (12, 13). While mobile connections in India crossed the one billion mark (14), only 25 per cent of rural households are digitally literate (8) and only 42 per cent of the mobile connections hail from rural pockets (14). While there is a consensus on how digitalisation has improved the efficacy of the healthcare system, the beneficiaries are often the ones who possess the skills to use digital technologies. Those with poor digital skills are sometimes adversely affected to the extent of deprivation of certain essential services. The idea of health as a human right involves four essential elements, viz. availability, accessibility, acceptability and quality (15). Of the four, “accessibility” is a key concern in terms of the digitalisation of healthcare services. By virtue of health being a human right, any factor that deters a person from attaining this right demands measures to ensure equitable access. The problem of individuals on the disadvantaged side of the digital divide essentially involves an accessibility-related violation of the right to health at two levels, i.e., the individuals who do not have the devices to access healthcare services digitally and the individuals who have the devices but are not competent enough to access healthcare services digitally.

With digital marginalisation emerging as a decisive factor for health (in)equity in an evolving digitalised healthcare landscape, research should not only focus on advancing technology but the human aspects of applying these technologies. While most studies reiterate the need for digital inclusion, the lived experience of the digitally marginalised is seldom emphasised. Understanding how digital marginalisation overlaps with other disadvantages to induce poor health outcomes for the marginalised is critical in addressing health inequity. This study focuses on how the digitalisation of healthcare services affects the digitally marginalised and how the intersection of digital marginalisation with other disadvantages results in compounded health inequity for vulnerable groups. Addressing the digitalisation of healthcare services from a human rights perspective, this study employs a case study approach and argues that the digitalisation of medical services, while essential and beneficial, should be made with caution not to marginalise vulnerable sections.

## Method

Since this study aimed to plot the experiences of a subset of the population that are outliers in the context of the phenomenon inquired, a qualitative approach, using case studies, was employed. To address the specific research question, i.e., how certain sections of society are marginalised due to the increasing digitalisation of healthcare services, illustrative cases are explored. Illustrative case studies explain a scenario or occurrence, delineate the events taking place, and elucidate the reasons behind its unfolding (16). They are particularly useful in elucidating the social processes involved in a phenomenon. The data was gathered using observational methods and self-reported narratives of the individuals/cases. The use of observational methods to supplement narratives was used with the aim of “seeing what people actually do” instead of just listening to “what they say they do” (17, 18). Besides, the observations from the service provider aspect help in understanding how the problem could effectively be addressed.

To address the impact of digitalisation on the digitally marginalised specifically, two older adults from a rural setting in Tamil Nadu, who were registered patients of a Filariasis Management Clinic at the ICMR-Vector Control Research Centre in the neighbouring Puducherry UT, where the authors are affiliated, were identified for the case study based on preliminary observations. The selection of the cases was based on the principles of illustrative case study, which demands the selection of a small number of cases with a detailed description of the phenomenon, its causes and effects (19). While multiple cases of digital marginalisation and consequent difficulties in availing healthcare services were presented in the clinical setting where the authors are affiliated, the cases narrated in this study were chosen based on the significant impact digital marginalisation had on healthcare prospects. The patients chosen for this study were referred to a tertiary care hospital for medical care, and the difficulties they faced while trying to access the digitalised healthcare services were observed by the authors and notes were taken at each stage. The observations, paired with narratives from the patients explaining how the digitalisation of healthcare service delivery was impacting their ability to access healthcare service, constitute the data for the study. The narratives of the patients were recorded after they were able to access the healthcare service. The narratives were then analysed for emerging themes using an inductive approach (20) to understand the experiences of the patients in navigating through digitalised healthcare services. The findings from the observations and the narratives are triangulated (21) and discussed in the context of digital marginalisation in healthcare service delivery.

### Findings from case observations

The observations were pertaining to the underlying circumstances leading to the event and its consequences on the health prospects of the patient. This section briefly discusses the case observations made by the clinician.

#### Observations from case 1

Case 1 of this study was a 60-year-old male with no formal education and job, hailing from a rural area, suffering from grade III lymphedema, which limited his mobility. The family comprised the patient's wife, a son and a daughter, who were all only having primary education. Upon examination of the case history, it was found that the patient had been evaluated earlier in a tertiary care hospital and was under follow-up there. The tertiary care hospital had a mobile phone-based appointment system for investigations, which allocated the date of appointment with a centralised system that communicated the appointment to the patient through text message. However, the patient did not own a mobile phone, and hence, he had given the mobile number of a friend for the purpose, who only had the knowledge to make calls but not manage text messages. The patient was subject to first-level digital marginalisation, as he had no access to the digital technology required to secure an essential service. The friend of the patient was subject to second-level digital marginalisation since he had poor knowledge of digital technologies, which led to sub-par use of the technology, resulting in missing out on an essential service. Consequently, they were both facing clearcut inequities in the benefits derived from ICT. This meant that the patient missed out on the message sent from the hospital and thereby missing out on the diagnostic tests prescribed. This is an unambiguous case where technology induces inequity in access to healthcare services. As technology permeates everyday lives and essential services of society, those who have no access and poor skills in ICT will be facing ICT-induced inequities, i.e., the third level of the digital divide permeates the digitally marginalised in society (22).

#### Observations from case 2

Case 2 of this study was the instance of a referral from the Filariasis Management Clinic to a tertiary care hospital. The patient was a 72-year-old male farmer with no formal education, suffering from filarial lymphedema, hailing from a distant village. The family consisted of the patient, his wife, and four children who were married and settled with their respective families. He developed a right earache, and on evaluation, a growth was detected in the ear canal. He was referred to a tertiary care government hospital for further evaluation, where a CT scan was advised and was told that the date would be sent to his mobile phone as a text message. The patient had limited skills in using a mobile phone with only the knowledge to make phone calls but not text messaging. The appointment date was conveyed from the tertiary care hospital via text message in English language. The patient had poor knowledge of handling text messages and was unable to read English, adding to the already difficult situation of poor digital knowledge. Since unfamiliar with accessing text messages, the patient missed out on the appointment. The patient came back to the clinic and expressed his agony, as he had to get another appointment, which further delayed the delivery of the healthcare service. Through an informal arrangement with the help of the staff at the healthcare centre, who informed the date of the appointment over a phone call, the patient was able to attend the next appointment.

These two cases depict how the digitalisation of healthcare services, despite its benefits, can adversely impact the digitally marginalised. While digitalised appointments for diagnostic tests in public hospitals can mitigate the problem of allocating resources effectively and eliminating preferential treatment, this case shows how relatively small tasks like securing a health service appointment can be challenging to the digitally marginalised.

While this section explains the ‘process’ of digital marginalisation in healthcare service delivery, the following section explains the practical difficulties and adverse impact experienced by the patients.

### What it means to be digitally marginalised: findings from the narratives

This section reflects how the patients navigated through the digital marginalisation experiences. In order to understand the experience of the digitally marginalised in accessing healthcare services, the narratives of the patient were recorded and analysed for emerging themes. The data thus generated yielded the following themes:

#### Theme 1: the physical and financial loss due to digital marginalisation in healthcare

Despite free healthcare access provided through public sector hospitals, healthcare remains inaccessible to many due to factors like out-of-pocket expenditure and poor socioeconomic status (23). The cases considered in this study involved individuals from rural areas who had to travel to a distant location in order to avail of healthcare services. Though the health care service is free, the travel incurs a cost to the patients. In one of the cases, after missing out on the first appointment for the CT scan, the patient started visiting the hospital every week to ensure that his appointment was not missed, incurring additional financial burden.

The process went on for two months. I used to go to the hospital weekly to make sure that I did not miss another appointment and get my treatment done. I constantly enquired about my appointment, as I had a lot of confusion. (Case 1)

This did not just incur a financial burden but also created physical difficulties. Both patients were older adults having lymphedema. Travelling and standing for longer duration are difficult for lymphedema patients. In Case 1, the patient was travelling to the location of the hospital multiple times just to make sure that he did not miss the appointment. In Case 2 as well, there were logistical difficulties as the patient was from a village and had to travel by public transport system to the hospital. After missing out on the first appointment, he had to make additional trips to the hospital to get a new appointment.

I had no idea that I had been given an appointment for my CT scan. I came to know about it after someone told me. I had to travel to the hospital again to get the appointment in an alternate slot. (Case 2).

This inconvenience due to lack of access and poor knowledge of digital technology is an example of digitalised healthcare failing to serve the purpose it was designed for. While technology can systematise workflow and bring in efficacious service delivery, the underlying assumption of digitally literate service seekers is a premise that needs to be cautiously approached in most developing nations. In this case, this digital marginalisation is found to be incurring additional financial burden and positing logistic and physical inconvenience for patients which needs attention. However, digitalisation *per se* is not the problem here, but its prudent use to ensure that digitally marginalised are provided additional support is essential.

#### Theme 2: sense of digital marginalisation and its impact on health-seeking behaviour

People hailing from poor socio-economic backgrounds and marginalised communities are often subject to health inequity by virtue of the very social system they are part of (24). The sense of marginalisation is often prevalent among individuals while approaching healthcare facilities, sometimes even created by poor doctor-patient relationships (25). Digitalisation, despite its benefits, can sometimes induce a sense of marginalisation for the digitally illiterate and can have implications on the health-seeking behaviour of individuals. The inability to avail of health services due to incompetent digital knowledge exerts a sense of marginalisation on the patients, and they tend to avoid medical needs unless extremely necessary to avoid the hassle associated with digital appointments.

After missing the first appointment, I told them that I am not good with text messages. A staff scolded me for missing out on the appointment and asked me not to miss out on the next appointment. (Case 2).

Persons who hail from the marginalised sections experience a double burden if they are not digitally literate. The lack of digital know-how makes it even more difficult for them to access the already difficult-to-access healthcare services. This can make them reluctant to seek medical services, particularly follow-ups.

I had to travel multiple times from my village to the hospital for this one appointment. I am old, and there is no one to help me every time I travel. Now, I only think of consulting a doctor in town only when absolutely essential. (Case 1).

The perception of being lesser citizens due to poor digital literacy can affect the perception of the marginalised sections towards the healthcare system. The public sector healthcare landscape, which is already plagued by poor service delivery and lack of trust and adherence by rural masses (26), can be further aggravated by digital marginalisation.

I had to suffer a lot because I was not able to manage the information provided through the phone. It is not possible for people like me to learn to read [text] messages at this age. I would prefer to go to a place without such arrangements. (Case 2).

The digitalisation of healthcare service delivery has proven to be exceptional in facilitating service delivery in many parts of the world, but there are individuals at the fringes of society who are not fortunate enough to access and/or master digital technologies. This can impact their belief and trust in the healthcare delivery system, impacting their healthcare-seeking patterns. The cases presented in this article are illustrative in purpose; the actual impact of digitalisation on healthcare marginalisation is a problem that goes beyond a few cases. Many sections of society with overlapping disadvantages like illiteracy, poverty, frailty and chronic diseases experience the brunt of the digitalisation of healthcare. In the cases presented here, factors like rural domicile, age, chronic disease, education and poor socioeconomic conditions were already intersecting to induce disadvantages for the patients and the lack of digital literacy compounded with these disadvantages adversely impacting their healthcare prospects, which demands alternative measures.

## Discussion

As humanity continues to advance technologically, it is essential not to leave behind people who are digitally marginalised (27). Digitalisation of healthcare services, including telehealth, digitalised appointments and online consultations, are designed to make healthcare services cost-effective, efficacious and accessible. However, at least in some cases, the basic premise that determines the success of digitalised healthcare services, i.e., digital literacy, is still a work in progress rather than a goal achieved. This study has presented two such cases, where patients with a chronic illness hailing from rural areas were unable to adhere to digitalised healthcare service appointments. Similarly, there are multiple sections of society that remain outside the scope of digitalised healthcare. Such cases need to be taken into consideration while advancing digitalised healthcare. Medical institutions depending heavily on digital services should have alternate mechanisms to bridge the ‘digital divide’ that affects the digitally marginalised. The expansion of digitalised healthcare should, hence, be mindful of being inclusive wherever possible and also implement alternative mechanisms to advance equitable health service delivery for those with limited digital access and literacy.

The cases from this study illustrate how digital marginalisation can delay healthcare, promote discrimination, and induce financial and physical challenges for the digitally marginalised. The sense of being left behind in the digital world is similar to any other form of social isolation. In contexts where digital marginalisation is a possibility, there should essentially be alternate mechanisms to ensure that poor knowledge of ICT platforms does not transform into another determinant of ill health. In such scenarios, having dedicated helpdesks for the “digitally unskilled” in major tertiary care hospitals is desirable. NGOs and voluntary organisations can play a significant role in setting up helpdesks that help individuals in need of assistance in availing of digitalised healthcare services. Encouraging patients to have a caretaker with the knowledge and skills in digital technologies may be possible in some scenarios. For instance, the Filariasis Management Clinic, where the authors serve, have been practising telemedicine services with the support of digitally aware caretakers of patients. Also, the staff spend considerable time imparting knowledge and skills in digital technology to patients and caregivers. However, digital education need not always be a feasible strategy for tertiary care hospitals that are burdened with caseloads. Another area which requires attention is training the health care workers and field staff in digital tools. Nurses can play a vital role as educators and promoters of digital technologies, guiding patients, especially those who require chronic care, through the complexities of the digital health landscape. Leveraging the support from family and local healthcare providers can enable the digitally marginalised to access digital technologies, thereby bridging health inequities. This should be combined with the adoption of user-friendly technology that addresses challenges to the use of digital technology, like low literacy and the physical aspects of ageing (28). Bridging the digital divide is of great significance for the sections of society that are already marginalised in terms of healthcare, like communities endemic to neglected tropical diseases like filariasis.

### Limitations

The study has taken into consideration only two cases, as the aim was to track the impact of digital marginalisation on the healthcare-seeking process of the participants in the natural course of events.

## Conclusion

Digital technology was conceived as an equaliser with the potential to accelerate the efforts of health equity at the global level (29). While digitalisation and digital technologies can improve access, utilisation and service delivery, they can also exacerbate existing disparities and create barriers for those who lack digital literacy or access to technology. Addressing digital health innovations from a health equity perspective is essential to ensure fair and equitable digital health advancements (30). The cases presented in this article illustrate how individuals from marginalised communities undergo further marginalisation in healthcare due to poor/no digital literacy. These challenges underscore the importance of addressing the digital divide and ensuring equitable access to healthcare for all. Policy interventions rarely address the digital marginalisation aspect of health (in)equity. With the exponential digitalisation of healthcare globally, policies need to be informed of the impact of digital marginalisation on health equity. The implications are applicable not just for the “digitally developing” nations but the “digitally developed” too, as digitalisation is not a binary expression of the digitally literate and illiterate but a “spectrum” where people possess different degrees of expertise in embracing digitalised services (6). This necessitates comprehensive interventions and policies suiting the specific needs of people at different levels of this spectrum ranging from digital literacy efforts to the development of accessible technologies. Hence, efforts to bridge the digital divide must go beyond simply improving digital literacy but should involve implementing targeted interventions and support systems so that we can work towards a future where everyone, regardless of their digital literacy or access to technology, can access quality healthcare services.

## Data Availability

The datasets presented in this article are not readily available because the data of this study include personal information from which the identities of the participants can be assumed. Requests to access the datasets should be directed to vijeshvcrc.icmr@gmail.com.
